# A Letter to Our Reviewers— the Core of *Pediatric Quality and Safety*

**DOI:** 10.1097/pq9.0000000000000795

**Published:** 2025-03-05

**Authors:** Richard J. Brilli, Richard Eugene McClead, Ryan S. Bode, Robert Gajarski, David C. Stockwell

**Affiliations:** From the * Department of Pediatrics, Nationwide Children’s Hospital, The Ohio State University College of Medicine, Columbus, Ohio; † Johns Hopkins Children’s Center, Baltimore, Md.; ‡ Department of Anesthesia and Critical Care Medicine, Johns Hopkins University School of Medicine, Baltimore, Md.

Ten years ago, we embarked on a journey to create a medical journal focused exclusively on quality improvement and patient safety in the pediatric community. Our mission was to create “an international, peer-reviewed, open-access, online periodical dedicated to providing healthcare professionals a forum to disseminate the results of quality improvement and patient safety initiatives that impact the lives of children from fetus to young adulthood.”

With our publisher’s support, Wolters Kluwer-LWW, *Pediatric Quality and Safety* (PQS) was born and, in September 2016, published its first issue. This initial issue featured commentaries from the chief executive officers of 2 children’s hospitals—reflecting their strong support for quality improvement and patient safety and the importance of a journal, such as PQS, to disseminate and publicize that work. Since then, PQS has received 1,246 original manuscripts, published 687 papers, and is now indexed on all major indices. It received its first impact factor (1.1) in June 2023, which increased to 1.2 last year. This result is an outstanding achievement, considering that fewer than 15% of the 30,000 published medical journals receive an impact factor.

Although most PQS authors are from the United States, we receive many papers from other regions, including Canada, Europe, Asia, Africa, and Australia. The PQS website receives over 6,000 views each month. Seventy percent of website visitors are from the United States; however, 20% are from the United Kingdom, India, Canada, Australia, and China.

*Pediatric Quality and Safety* would not be possible without the strong support of a pediatric quality improvement and safety community that serves as peer reviewers for the Journal. Each published manuscript has at least 2 peer reviewers with quality improvement and subject matter content expertise. Journal peer reviewing can be a “thankless job,” and while the recognition for the time and effort required to complete a thorough and constructive critique is limited, it is essential for any journal. To this end, we are working with our publishers to develop a CME component for journal reviews, similar to other larger journals such as *NEJM* and *Pediatrics*.

Much of our Journal’s success has derived from the more than 2,000 PQS peer reviewers and our superb Editorial Board, who have given their time and expertise to help us publish cutting-edge and pragmatic quality improvement work.

We applaud your dedication and THANK YOU for your commitment to the core component of our journal—high-quality peer review.

Those reviewers who have reviewed 10 or more manuscripts are listed in the table, ordered by the number of completed reviews.

**Table:** 

Thomas Bartman, MD, PhD	Onsy Ayad, MD	Vicki Montgomery, MD
David C. Stockwell, MD, MBA	Loren Berman, MD, MHS	Brendan Boyle, MD
Sandra Spencer, MD	Raina Paul, MD	Jon Wispe, MD, MD
Colleen Briana Bertoni, MD, MBOE	Brian Joy, MD	Kristen M. Crandall, MSN, RN, CPN
Ryan Bode, MD	Uday Chalwadi, MD	Munish Gupta, MD
Julie Samora, MD, PhD	Amy Louise Billett, MD	Anthony Alexander Sochet, MD, MSHS
Jayant Deshpande, MD, MPH	Vijay Srinivasan, MD	Rosalyn Stewart, MD, MS, MBA
Gary Frank, MD	Elizabeth Mack, MD, MS	Lennox Huang, MD
Joshua C. Uffman, MD	Ryan Coller, MD, MPH	Olivia Lund Hoffman, MD
Carol Kemper, PhD, RN	Jahnavi Valleru, MS, MHA	Courtney Nelson, MD
Lloyd Provost, PhD	Steven Allen, MD	Laurel Moyer, MD
Rahul Shah, MD, MBA	Kelly C. Sandberg, MD, MSc	Daniel J. Scherzer, MD
Jason Newland, MD, MEd	Shannon H. Baumer-Mouradian, MD	Kevin J. Little, MD
Robert Gajarski, MD	Anne Stack, MD	Derek Wakeman, MD
Sandip Godambe, MD, PhD	James Hoffman, PharmD, MS	Anthony Lee, MD
Christopher Dandoy, MD	Anup Patel, MD	Stephen Andrew Spooner, MD, MS
Todd Karsies, MD	Eugenia K Pallotto, MD	Tensing Maa, MD
Mike Fetzer, BSISE	Jeffrey Lutmer, MD	Anu Subramony, MD, MD, MBA
Michael F. Wells, MD	Jamie Macklin, MD	Jack Stevens, PhD
Shawn Rangel, MD, MSCE	Kathleen E. Walsh, MD, MS	Daniel Ehrmann, MD, MS
Thomas Taghon, DO	Jonathan Thackeray, MD	Ashley Cooper, MD
Gail Bagwell, DNP	Roopali Bapat, MD	Anthony Sochet, MD, MSHS
W. Charles Huskins, MD, MSc	Allison King, PharmD	Christopher Bonafide, MD, MSCE
Joshua Watson, MD	Claudia Algaze, MD, MS	Robert Angert, MD
Elizabeth D. Allen, MD	Jennifer Melvin, MD	
Daniel Hyman, MD	Brian Coley, MD	
Edward G. Shepherd, MD	Ryan Breuer, MD	
Beverly Brozanski, MD	Jeffrey B. Anderson, MD, MPH, MBA	
Lori Rutman, MD, MPH	Berkeley Bennett, MD	
Paul Sharek, MD, MPH	Paul C. Mullan, MD, MPH	
Beth Emerson, MD	Trisha Marshall, MD	
Lamia Soghier, MD	Deena Berkowitz, MD, MPH	
Mark Del Beccaro, MD	Janet Berry, DNP, RN, MBA, NEA-BC, CNOR	
Derek S. Wheeler, MD, MMM, MBA	Dmitry Tumin, PhD	
John Chuo, MD	Megan M. Letson, MD, MEd	
Tetsu (Butch) Uejima, MD	Christina L. Cifra, MD, MS	
William Matthew Linam, MD, MS	Peter Lachman, MD, MPH, FRCPCH	
Thomas J. Caruso, MD	John Mahan, MD	
Ramachandra Bhat, MD	Kerry Rosen, MD	
Leigh Anne Bakel, MD	Jacqueline B. Corboy, MD, MS	
Dane Snyder, MD	Theresa R. Grover, MD	
Anthony Piazza, MD	Jeffrey Hoffman, MD	
Kris Jatana, MD	Randal Olshefski, MD	
Kelly Kelleher, MD, MPH		
Marlene Miller, MD, MSc		

